# Obstructive Sleep Apnea and a Comprehensive Remotely Supervised Rehabilitation Program: Protocol for a Randomized Controlled Trial

**DOI:** 10.2196/47460

**Published:** 2023-09-18

**Authors:** Jakub Hnatiak, Lujza Zikmund Galkova, Petr Winnige, Ladislav Batalik, Filip Dosbaba, Ondrej Ludka, Jan Krejci

**Affiliations:** 1 Department of Rehabilitation University Hospital Brno Brno Czech Republic; 2 First Department of Internal Medicine - Cardioangiology, St Anne´s University Hospital Faculty of Medicine Masaryk University Brno Czech Republic; 3 Cardiovascular Sleep Center of the First Department of Internal Medicine - Cardioangiology, St Anne´s University Hospital Brno Czech Republic; 4 Department of Public Health Faculty of Medicine Masaryk University Brno Czech Republic; 5 Department of Internal Medicine, Geriatrics and Practical Medicine University Hospital Brno Brno Czech Republic

**Keywords:** obstructive sleep apnea, telerehabilitation, telemonitoring, CPAP, apnea-hypopnea index, telehealth, telemedicine, sleep, respiratory, home based, rehabilitation, RCT, randomized controlled trial

## Abstract

**Background:**

Obstructive sleep apnea (OSA) is characterized by recurrent, intermittent partial or complete obstruction of the upper respiratory tract during sleep, which negatively affects the patient's daily quality of life (QoL). Middle-aged and older men who smoke and have obesity are most at risk. Even though the use of continuous positive airway pressure (CPAP) during sleep remains the gold standard treatment, various rehabilitation methods, such as exercise, respiratory therapy, myofunctional therapy, and nutritional lifestyle interventions, also appear to be effective. Moreover, it is increasingly recommended to use alternative or additional therapy options in combination with CPAP therapy.

**Objective:**

This study aims to evaluate if a comprehensive home-based, remotely supervised rehabilitation program (tele-RHB), in combination with standard therapy, can improve OSA severity by decreasing the apnea-hypopnea index (AHI); improve objective parameters of polysomnographic, spirometric, anthropometric, and body composition examinations; improve lipid profile, maximal mouth pressure, and functional capacity tests; and enhance the subjective perception of QoL, as well as daytime sleepiness in male participants with moderate to severe OSA. Our hypothesis is that a combination of the tele-RHB program and CPAP therapy will be more effective by improving OSA severity and the abovementioned parameters.

**Methods:**

This randomized controlled trial aims to recruit 50 male participants between the ages of 30 and 60 years with newly diagnosed moderate to severe OSA. Participants will be randomized 1:1, either to a 12-week tele-RHB program along with CPAP therapy or to CPAP therapy alone. After the completion of the intervention, the participants will be invited to complete a 1-year follow-up. The primary outcomes will be the polysomnographic value of AHI, Epworth Sleepiness Scale score, 36-Item Short Form Health Survey (SF-36) score, percentage of body fat, 6-minute walk test distance covered, as well as maximal inspiratory and expiratory mouth pressure values. Secondary outcomes will include polysomnographic values of oxygen desaturation index, supine AHI, total sleep time, average heart rate, mean oxygen saturation, and the percentage of time with oxygen saturation below 90%; anthropometric measurements of neck, waist, and hip circumference; BMI values; forced vital capacity; forced expiratory volume in 1 second; World Health Organization’s tool to measure QoL (WHOQOL-BREF) score; and lipid profile values.

**Results:**

Study recruitment began on October 25, 2021, and the estimated study completion date is December 2024. Analyses will be performed to examine whether the combination of the tele-RHB program and CPAP therapy will be more effective in the reduction of OSA severity and improvement of QoL, body composition and circumferences, exercise tolerance, lipid profile, as well as respiratory muscle and lung function, compared to CPAP therapy alone.

**Conclusions:**

The study will evaluate the effect of a comprehensive tele-RHB program on selected parameters mentioned above in male participants. The results of this intervention could help the further development of novel additional therapeutic home-based options for OSA.

**Trial Registration:**

ClinicalTrials.gov NCT04759456; https://clinicaltrials.gov/ct2/show/NCT04759456

**International Registered Report Identifier (IRRID):**

DERR1-10.2196/47460

## Introduction

### Background

Obstructive sleep apnea (OSA), the most common type of sleep-related breathing disorder [1], is characterized by recurrent, intermittent partial or complete collapse or obstruction of the upper respiratory tract during sleep [2]. Risk factors for its development include male gender, middle and older age, tobacco use, and obesity [3,4]. The high worldwide prevalence and increased risk of morbidity and mortality, especially due to cardiovascular diseases, make OSA a serious health-related global problem [5-7]. In addition, OSA can negatively affect the patient’s quality of daily life (QoL) [8]. For instance, excessive daytime sleepiness; impaired cognitive functions; work performance; ability to concentrate; or the occurrence of depression, headaches, snoring, and nocturnal urination; or disharmony in social interactions are particularly limiting [9,10]. The gold-standard treatment for OSA is continuous positive airway pressure (CPAP). The aim of CPAP therapy is to reduce the frequency of apneic and hypopneic pauses during sleep [11,12]. It is increasingly recommended to use alternative or additional therapy options in combination with CPAP [13]. These options include lifestyle and nutrition interventions, physical activity, pharmacotherapy, and behavioral therapy [13]. A recent meta-analysis has shown that a positive effect of exercise therapy, together with a reduction-diet intervention, leads to a significant reduction in the severity of OSA [14]. Based on several meta-analyses, the severity of the disease could be improved either by adjusting to a more nutritional lifestyle [14-16] or by implementing a physical intervention [17,18]. A significant reduction in the apnea-hypopnea index (AHI) was also achieved by training orofacial muscles with oropharyngeal exercises (ie, myofunctional therapy) [19]. According to Camacho et al [19], myofunctional therapy could represent an additional part of OSA therapy. Even the standard treatment representing CPAP therapy or mandibular advancement devices should also include exercise training [20]. A recent meta-analysis also showed a positive effect of respiratory therapy and respiratory muscle training elements in influencing OSA [21]. However, the authors stated that further research is needed to confirm the effect of additional therapeutic options already mentioned [12,15,17-19,21].

Aerobic and respiratory muscles training, oropharyngeal exercises, and regimen recommendations concerning lifestyle, nutrition, and behavior changes are individual components of the comprehensive home-based remotely supervised rehabilitation (tele-RHB) program conceived in this study. No recent literature has been found that deals with the effects of a similar and thus complex remotely supervised intervention. The absence of such an intervention in the context of OSA, as a possible therapeutic option for additional at-home training in combination with CPAP therapy, was the reason for conducting our research.

### Objective

This study protocol describes the first-ever randomized controlled trial to solely evaluate the effectiveness of the tele-RHB program among male adults with moderate to severe OSA. This study aims to assess whether completing a comprehensive telerehabilitation program and CPAP therapy positively affects the severity of OSA, QoL, body composition and circumferences, exercise tolerance, lipid profile, and respiratory muscle and lung function. Our hypothesis is that a combination of tele-RHB program and CPAP therapy will be more effective in the reduction of OSA severity and improvement of QoL, body composition, exercise tolerance, lipid profile, as well as respiratory muscle and lung function, compared to CPAP therapy alone.

## Methods

### Study Design

**Figure 1:**
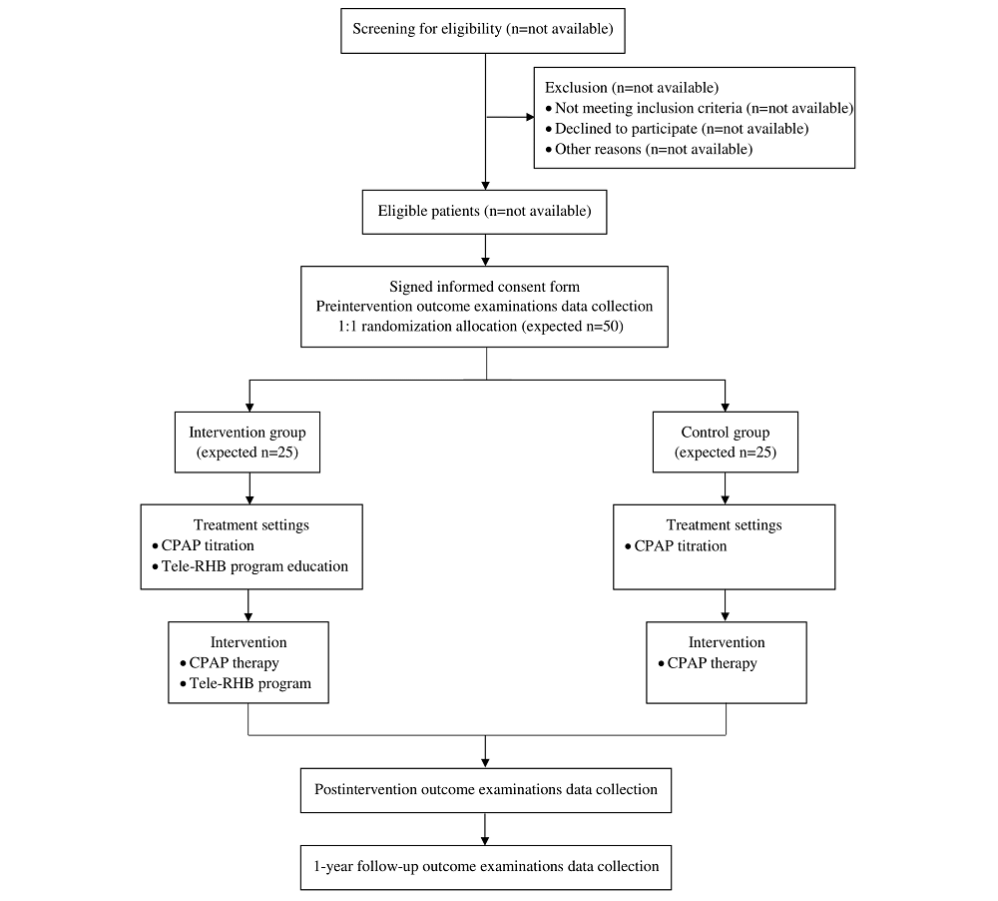
Flow diagram of the study protocol. CPAP: continuous positive airway pressure; tele-RHB: comprehensive home-based remotely supervised rehabilitation.

This study is a 2-arm parallel single-blind randomized controlled trial (registered on ClinicalTrials.gov: NCT04759456), with the per-protocol analysis investigating the effect of the tele-RHB program among 50 male patients with newly diagnosed OSA indicated to CPAP therapy. Patients were recruited from the Sleep Centre of the International Clinical Research Center of St Anne's University Hospital in Brno (FNUSA-ICRC). After providing informed consent, the patients will be randomized to the 12-week tele-RHB program along with CPAP therapy; 25 patients will be randomized to the intervention arm, and 25 will be in the control arm (CPAP therapy alone) in a 1:1 ratio, following the methods described below (Figure 1).

### Ethical Considerations

The study protocol follows the Declaration of Helsinki and received approval from the Ethics Committee of the University Hospital Brno in the Czech Republic (06-090920/EK). Participation in the study will be voluntary, without any compensation or remuneration, and participants will be allowed to withdraw from the study at any moment without providing a reason.

All participants will provide written informed consent. This informed consent form provides details about the purpose of the study, requirements for study participation, randomization process, personal data processing, storage and protection process, and contact information for research team staff. The original informed consent allows the secondary analysis of obtained data without additional consent.

All participants’ obtained data will be pseudonymized and deidentified; only an authorized person of the study research team will be able to identify the participants on the basis of personal data. The obtained data will be published anonymously, and no report or publication will contain any data that could lead to the participants’ identification.

### Inclusion Criteria

Participants will be eligible if they are male patients, aged 30-60 years, have newly diagnosed OSA with an AHI ≥15 episodes per hour, are indicated to CPAP therapy, and are interested in our adjunct tele-RHB program.

### Exclusion Criteria

Participants are excluded if they have another type of OSA treatment, chronic corticosteroid therapy or long-term oxygen therapy; they are also excluded if they have at least one of the following health problems: central sleep apnea, chronic obstructive pulmonary disease III or IV, severe pulmonary hypertension, New York Heart Association III or IV, acute coronary syndrome, heart failure, ejection fraction <40%, electrocardiographic-documented heart arrhythmia, severe heart valve disease, cerebrovascular disease, psychiatric disease, and physical disability; in addition, they will be excluded if they do not cooperate during the intervention or do not follow the tele-RHB program and CPAP therapy protocol.

### Recruitment, Enrollment, and Procedure

The patients who meet the inclusion criteria will be recruited from the Sleep Centre of the FNUSA-ICRC. The details of the study overview are shown in Table 1. On the first visit, patients will undergo polysomnography (PSG), blood sampling for lipid profile, and completion of the Epworth Sleepiness Scale (ESS) with a known score. Those who are found to have moderate to severe OSA will be contacted for further intervention alongside the standard CPAP therapy. These patients will be verbally informed and will receive an information leaflet about the ongoing study of the 12-week tele-RHB program. Then, they will complete anthropometric and spirometric examinations, measurements of maximal inspiratory mouth pressure (MIP) and maximal expiratory mouth pressure (MEP) as well as body composition, 6-minute walk test (6MWT), and QoL questionnaires in the Cardiovascular Rehabilitation Clinic at the University Hospital Brno (CRC-UHB). After that, during the interview with a physiotherapist, participants will receive detailed and comprehensive information regarding their possible participation in the study. Then, potential participants will be given at least 24 hours to decide before signing the informed consent.

On the second visit, patients who agreed to participate in the study will sign the informed consent form and will be then randomized 1:1 to the intervention or control group.

On the third visit, participants of both groups will undergo individual titration of CPAP therapy at the Sleep Centre of the FNUSA-ICRC. The intervention group will subsequently receive education about the tele-RHB program, along with theoretical and practical instruction on its individual components in the CRC-UHB. Participants of the intervention group will also receive a training brochure containing information needed for the tele-RHB program with remote supervision by a physiotherapist.

After the completion of the 12-week tele-RHB program, on the fourth visit, participants of both groups will undergo the same examinations and measurements as those conducted at the baseline, at the Sleep Centre of the FNUSA-ICRC and in the CRC-UHB.

After 1 year from the 12-week tele-RHB program’s completion, on the fifth visit, participants of both groups will undergo the same examinations and measurements as they did at the baseline in both health facilities.

**Table 1:** An overview of the study flow.

Timeline	Preintervention outcome examination (0-7 days)			Treatment settings (8-28 days)		Intervention (12 weeks)		Postintervention outcome examination (1-5 days after)		Follow-up (1 year after)
	First visit	Second visit	Third visit		N/A^a^		Fourth visit		Fifth visit	
Screening	✓									
Signed informed consent form		✓								
Interview	✓									
Randomization		✓								
Polysomnography	✓						✓		✓	
Epworth Sleepiness Scale	✓						✓		✓	
Lipid profile	✓						✓		✓	
Anthropometry	✓						✓		✓	
Spirometry	✓						✓		✓	
Maximal mouth pressures	✓						✓		✓	
6-minute walk test	✓						✓		✓	
Body composition	✓						✓		✓	
Questionnaires	✓						✓		✓	
CPAP^b^ titration			✓							
Education			✓							
Teleconsultations					✓					

^a^N/A: not applicable.

^b^CPAP: continuous positive airway pressure.

### Randomization

Participants will be randomized 1:1 to the intervention and control group by a noninvestigating member of the research group in the CRC-UHB with the help of the website “randomization.com” [22] into 6 blocks of 8 and 1 block of 2 participants. Participants and caregivers will be instructed not to comment on their ongoing therapy. The outcome assessor will be blinded. Physiotherapists who educate the intervention group participants about the tele-RHB program and who carry out regular teleconsultations during this program are the only unblinded members of the research group.

### Measures

#### Measurements and Examinations

All participants will take part in baseline, postintervention, and follow-up examinations. PSG will be performed by trained medical staff in accordance with recent guidelines [23]. The variables extracted from PSG will be AHI, oxygen desaturation index, supine AHI, total sleep time, average heart rate, mean oxygen saturation (SpO_2_), and the percentage of time with SpO_2_ saturation below 90%. PSG will be performed with Grael 4K PSG:EEG (Compumedics Limited). All of the above-mentioned data will be collected without the CPAP device in use.

Standardized questionnaires, including the 36-item Short Form Health Survey (SF-36), the World Health Organization’s tool to assess QoL (WHOQOL-BREF) [24], and ESS, will be filled in by all participants under the supervision of a medical specialist. The ESS questionnaire will be a tool for the participant’s subjective daytime sleepiness, and the SF-36 and WHOQOL-BREF questionnaires will assess the subjective perception of QoL.

Participants will undergo a bioelectrical impedance examination with InBody 370S (BridgePower Corp) to evaluate changes in body composition performed by trained medical personnel. Data on the participant’s sex, age, height, weight, and BMI will also be recorded. The monitored parameter will be the value of the percentage of body fat.

Participants will undergo a standardized 6MWT in accordance with American Thoracic Society guidelines to objectively assess their functional capacity by trained medical personnel [25]. The monitored parameter will be the distance in meters, which the participants will be able to walk in 6 minutes.

MIP and MEP will be measured with MicroRPM Respiratory Pressure Meter (Micro Direct Inc) and will be performed by a medical specialist based on recent recommendations for testing respiratory muscles [26,27].

Anthropometric parameters of the neck, waist, and hip circumference will be measured with a tape measure by trained medical staff. The current BMI value will also be calculated from actual body height and weight.

All participants undergo a standardized spirometric examination performed by a medical specialist with Spirobank II Basic (Medical International Research) in accordance with recommended procedures to objectively evaluate their lung function [28]. The parameters of forced vital capacity and forced expiratory volume in 1 second will be monitored.

All participants’ CPAP devices will be regularly remotely monitored by a Sleep Centre medical doctor to track CPAP compliance and adherence to treatment during their sleep-at-home conditions. Participants will use one of the following devices: AirSense 10 AutoSet (ResMed Inc) or Prisma 20A Auto CPAP (Löwenstein Medical Technology GmbH + Co. KG).

Participants will have laboratory blood tests with the intention of finding out the current values of their lipid profile. Values of the total cholesterol, triacylglycerols, high-density lipoprotein (HDL) cholesterol, low-density lipoproteins (LDL) cholesterol, and non-HDL cholesterol will be detected.

#### Primary Outcomes

Primary outcomes include changes from baseline in AHI, obtained from PSG; ESS score; SF-36 score; percentage of body fat; 6MWT distance; as well as MIP and MEP values.

#### Secondary Outcomes

Secondary outcomes include changes from baseline in oxygen desaturation index, supine AHI, total sleep time, average heart rate, mean SpO_2_, the percentage of time with oxygen saturation below 90%, obtained from PSG; neck, waist, and hip circumference; BMI values; forced vital capacity and forced expiratory volume in 1 second values; WHOQOL-BREF score; CPAP adherence; as well as total cholesterol, triacylglycerols, HDL cholesterol, LDL cholesterol, and non-HDL cholesterol values.

#### Intervention Group

Participants of the intervention group will undergo home-based 12-week tele-RHB program with regular teleconsultations via phone calls and emails at least 1-2 times a week with a physiotherapist. Teleconsultations will include telemonitoring and analysis of previously completed training sessions and the current course of the program; additionally, telecoaching for upcoming training sessions and motivation to continue the program will also be provided. Participants will also record the details of their completed training sessions during the intervention in a training diary and will regularly send it to the physiotherapist for evaluation. As previously stated, participants will receive a training brochure with a detailed description of the tele-RHB program and its components (Multimedia Appendix 1). Tele-RHB program will include nutrition, health-related lifestyle and behavior change recommendations, and a minimum of 5 times a week—each lasting 30 minutes—of moderate-intensity aerobic training; in addition, there will be 10 minutes of inspiratory and 10 minutes of expiratory muscle training with breathing devices (Threshold IMT and Threshold PEP) and 10 minutes of oropharyngeal exercise along with individually titrated CPAP therapy. After the completion of the 12-week tele-RHB program, participants should be motivated to continue performing this comprehensive rehabilitation program in home conditions during the 1-year follow-up, but without the regular remote supervision by a physiotherapist. This tele-RHB program was developed based on previous research or guideline recommendations for patients with OSA or cardiovascular diseases [6,29-36].

#### Control Group

Participants of the control group will undergo individually titrated CPAP therapy alone, throughout 12 weeks.

### Data Analysis

#### Statistical Analysis

TIBCO Statistica (TIBCO Software Inc) will be used for statistical analysis of the obtained data. Statistical analysis will be performed by a person blinded to the experimental conditions. The Shapiro-Wilk test will be used to assess the normality of the data obtained. To compare participants’ baseline and postintervention obtained data within the same group, a paired 2-tailed *t* test will be used for statistical analysis. When comparing the baseline and postintervention data of the participants between both groups, an unpaired *t* test will be used. The level of statistical significance will be set as *P*<.05. The acquired data will be securely collected on an encrypted external hard drive.

#### Sample Size

A power analysis was performed based on a previous study by Hupin et al [37], and the expected improvement in AHI was estimated to be approximately 8 (SD 10) episodes per hour at the end of the program versus the baseline. The number of participants in each of the two groups (n=25), necessary to achieve such an effect size, was calculated by statistical analysis at 80% statistical power with α=.05.

## Results

Study recruitment began on October 25, 2021, and the estimated study completion date is December 2024. Analyses will be performed to examine whether the combination of the tele-RHB program and CPAP therapy will be more effective in the reduction of OSA severity and improvement of QoL, body composition and circumferences, exercise tolerance, lipid profile, as well as respiratory muscle and lung function, compared to CPAP therapy alone.

## Discussion

OSA is a severe global problem [6,7,38], negatively affecting the QoL and its various aspects [8,38]. Furthermore, although CPAP therapy is currently considered the gold standard of treatment [38], adherence to its long-term use is low [39]. According to Aardoom et al [40], about 30% to 80% of patients with OSA are nonadherent to their CPAP therapy. However, CPAP adherence and therapy compliance can be improved by a telemedicine-based approach (ie, eHealth) containing regular teleconsultations via video calls [41]. According to Hnatiak et al’s [42] opinion, an additional therapy consisting of the implementation of the tele-RHB program could also improve the severity of OSA or even help with adherence to CPAP therapy. In addition, our comprehensive program, consisting of effective elements of rehabilitation, includes aerobic exercise training [17,18], oropharyngeal training [19], and respiratory muscle training [21], along with recommendations focusing on nutrition, lifestyle, and behavior changes [14-16].

The development of this tele-RHB program for the examined population was based on currently available knowledge. The comprehensive tele-RHB program was designed according to information about effective additional treatment options for OSA mentioned earlier. The study included only middle-aged and older male adults due to the risk of more frequent OSA occurrences in these individuals [3,4]. Thus, significant risk factors for the development of OSA also include middle [4] or older age, smoking [3], and obesity [3,4].

A possible limitation may be the lack of direct physiotherapist supervision during the remotely monitored intervention in a home-based setting. According to Hnatiak et al [42], regular communication of participants with physiotherapists during the intervention should help the standard course of the intervention. Thus, we assume that the completion of this intervention will produce interesting results that will help the further development of additional therapeutic options for OSA.

Our study intends to determine whether the completion of a tele-RHB program positively affects OSA in middle-aged and older men, and therefore, provide evidence for a possible recommendation of this tele-RHB program as an effective adjunctive treatment to CPAP therapy in male patients. The study will focus on the expected improvement in OSA severity, QoL, body composition, exercise tolerance, lipid profile, as well as respiratory muscles and lung function, resulting from the completed intervention.

## Appendix

Multimedia Appendix 1 Training brochure—detailed description of the Tele-RHB (comprehensive home-based remotely supervised rehabilitation) program.

## Abbreviations

6MWT: 6-minute walk test
AHI: apnea-hypopnea index
CPAP: continuous positive airway pressure
CRC-UHB: Cardiovascular Rehabilitation Clinic at the University Hospital Brno
ESS: Epworth Sleepiness Scale
FNUSA-ICRC: International Clinical Research Center of St Anne’s University Hospital in Brno
HDL: high-density lipoproteins
LDL: low-density lipoproteins
MEP: maximal expiratory mouth pressure
MIP: maximal inspiratory mouth pressure
OSA: obstructive sleep apnea
PSG: polysomnography
QoL: quality of life
SF-36: Short Form Health Survey-36
SpO_2_: oxygen saturation
Tele-RHB: comprehensive home-based remotely supervised rehabilitation
WHOQOL-BREF: World Health Organization Quality of Life-BREF

## Data Availability

The data sets generated and analyzed during this study are available from the corresponding author upon reasonable request.
