# Supplementary material for: Obstructive Sleep Apnea and a Comprehensive Remotely Supervised Rehabilitation Program: Protocol for a Randomized Controlled Trial
Source: JMIR Res Protoc. 2023 Sep 18;12:e47460. doi: 10.2196/47460 (PMC10546260; doi:10.2196/47460)
Supplement: Multimedia Appendix 1 [file resprot_v12i1e47460_app1.pdf]

Dear study participant,

On behalf of myself and the co-authors, I would like to thank you in advance for deciding to participate in our study of the impact of a remotely-supervised rehabilitation program for patients with newly diagnosed obstructive sleep apnea. The purpose of this brochure is to guide you through the entire twelve-week regular training program so that by completing it your illness will improve and alleviate the associated difficulties. The brochure can help you with regular exercise even after completing the study.

Before you begin

In the outpatient clinic of cardiovascular rehabilitation at the University Hospital Brno, you personally underwent education on obstructive sleep apnea and regimen recommendations for lifestyle changes, nutritional and behavioral changes, along with practical instruction focused on aerobic-endurance walking training, training of inspiratory and expiratory muscles using respiratory training devices and exercise of oropharyngeal muscles during a twelve-week program with remote monitoring by a physiotherapist.

With the physiotherapist, you have also agreed on the details of regular teleconsultations carried out at least once every 5 days by telephone or e-mail. Teleconsultations are an integral part of the entire program and serve primarily to telemonitor your previous training, telecoaching of next training or as your possible motivation to continue the training program. Your 12-week remotely-supervised rehabilitation program will consist of:

- regime recommendations - lifestyle changes, nutritional and behavioral changes,
- aerobic-endurance training,
- respiratory training of the inspiratory muscles,
- respiratory training of the expiratory muscles,
- exercise of oropharyngeal muscles,
- teleconsultations - telemonitoring, telecoaching and motivation.

Training program

Below is a detailed description of the individual components of your rehabilitation program. The information contained here was communicated to you by a physiotherapist during the initial education together with practical instruction. The purpose of this brochure is to keep this important information with you at all times, so that you can draw from it at any time during the program, and so that you are not entirely dependent on the regular teleconsultations with a physiotherapist.

Regime recommendations - nutritional changes

The nutritional change recommendations you should use to adjust your diet and eating habits are based on recommendations issued by the American Academy of Sleep Medicine (AASM) and the American Heart Association (AHA). The main purpose of these recommendations and the adherence to them is to reduce your body weight, especially fat mass, which can positively affect the severity and manifestations of obstructive sleep apnea. By following the recommendations during and even after completing the rehabilitation program, you can continue to positively influence not only your health, but even your entire life and its quality. The recommendations below are:

- relate the daily caloric intake to the observed value of the basal metabolic rate, taking into account the level of physical activity,
- observe a balanced daily caloric intake and expenditure,
- consume 3-5 similarly hearty meals regularly during the day,
- avoid consuming heavy meals in the evening, have the last meal 3-4 hours before going to bed,
- prefer a varied and quality diet daily, rich in important nutrients - carbohydrates (approx. 60% of daily intake), proteins (10-20%), fats (<30%), minerals, vitamins,
- consume vegetables daily (canned, dried, fresh, frozen), at least 250g
- consume fruit daily (canned, dried, fresh, frozen), at least 200g
- consume cereal and whole grain ingredients daily (from barley, brown rice, millet, oats, wholemeal bread, crackers, pasta), between 85-170 g,
- consume daily non-fat or low-fat dairy products, approximately 300 g,
- consume daily some of the protein sources (eggs, fish and poultry without skin, lean meat, legumes, nuts, seeds), at least 155 g,
- prefer polyunsaturated and monounsaturated oils (rapeseed, olive, safflower, sunflower,
- sesame oil), approximately 3 tablespoons / 30 g,
- reduce saturated fats, trans fats, salt, fatty meats, sweets, sugary drinks, whole milk products, coconut and palm oil, highly processed foods,
- be aware of the caloric values and composition of the food consumed,
- choose products with the lowest amounts of sodium, added sugars, saturated fats, trans fats and without partially hydrogenated (hardened) oils / fats.

Regime Recommendations - Lifestyle Changes and Behavioral Changes

Recommendations for lifestyle changes and behavioral changes relate to adjusting day to day and sleep patterns, influencing risk factors, alcohol consumption, smoking, medications affecting sleep and performing regular physical activity. Recommendations regarding changes in eating habits and weight reduction are described in more detail in the previous section of this brochure. The individual recommendations, based on recent expert sources, therefore represent:

- set and follow a regular daily routine - keep work and non-work activities, meals and physical activities at a similar time,
- avoid longer sleep during the day, a short afternoon "nap" lasting up to 30 minutes does not matter,
- follow your treatment and prescribed medications, or discuss with your doctor the medications and medications that affect your sleep (eg hypnotics, sedatives, muscle relaxants, antihistamines, opiates, antiemetics, antidepressants, neuroleptics, anticonvulsants, some beta-blockers, ACE inhibitors, theophylline, corticosteroids, nasal decongestants, amphetamines),
- do not smoke or at least reduce smoking as much as possible,
- avoid consuming caffeine and stimulants (coffee, green and black tea, cola drinks, energy drinks) 4-6 hours before going to bed,
- do not consume alcohol 4-6 hours before going to bed, preferably do not consume alcohol at all,
- set and maintain a regular sleep regime - have enough quality, regular, reasonably long and uninterrupted sleep, go to sleep and get up regularly at a similar time,
- observe proper sleep hygiene - minimize noise and light and have a suitable temperature (18-20 ^{o}C) during your sleep,
- do not spend the night or, on the contrary, do not sleep too much,
- sleep in a suitable position - avoid sleeping on your back if it causes you to have breath breaks.

Aerobic-endurance training

Regularly performed aerobic-endurance training is conceived as walking training. You will perform it in home conditions or in the vicinity of your home according to the initial

education by a physiotherapist and the parameters listed below - intensity, frequency and duration.

The intensity you will observe during your walking training is grade 13-14 according to the Borg scale of subjective perception of exercise intensity. The load set in this way should correspond to your subjective evaluation of walking training as "somewhat difficult".

You should observe the frequency and duration of walking training during the program with a frequency of at least 5 times a week and a duration of at least 30 minutes each.

Respiratory training of the inspiratory muscles

You will use the Threshold® IMT respiratory trainer at home to perform respiratory muscle training as part of your rehabilitation program. You will perform the training to the best of your abilities while adhering to its parameters below - load, frequency, duration, number of series, number of repetitions and conditions of its implementation.

The training load of the inspiratory muscles was set for you individually according to the examination of the maximum oral pressure of the inspiratory muscles (MIP), which you initially completed with a physiotherapist. Subsequent training with a respiratory trainer will be completed at 30% of the maximum value you achieved during the MIP examination. If you were not able to handle this set value during practical instruction by a physiotherapist for various objective or subjective reasons, you will complete the training with a lower value than the set 30% MIP. The physiotherapist told you the initial value that you will observe during the training of the respiratory muscles during the initial education.

You should adhere to the frequency and duration of the inspiratory muscle training of the rehabilitation program at a frequency of at least 5 times a week and the duration of each of them at least 10 minutes, provided that you have completed the set number of sets and repetitions below.

Each of your respiratory muscle training will consist of 5 series of 10 repetitions. Take a 1-minute break after each series.

Exercise your breathing muscles with the training device sitting upright and with the nose clip attached so that you inhale exclusively through your mouth through the Threshold® IMT and not through your nose during training. Inhale against the set resistance of the training device, but it should not represent your maximum inhale. Subsequent exhalation should take longer than inhalation, the approximate ratio is 2:1. Perform the respiratory training of the inspiratory muscles in an upright sitting position and, if possible, with a visual inspection for better performance of your training (eg a mirror).

Respiratory training of expiratory muscles

You will use the Threshold® PEP respiratory trainer at home to perform respiratory muscle training regularly as part of your rehabilitation program. You will also train the exhalation muscles according to your abilities, while adhering to its parameters listed below - load,frequency, duration, number of sets, number of repetitions and conditions of its execution. The settings of the exhalation muscle training with the Threshold® PEP respiratory trainer do not differ much from that for the inspiratory muscles, but for completeness we also present its complete settings below.

The training load of the exhaled muscles was set for you individually according to the examination of the maximum oral pressure of the exhaled muscles (MEP), which you initially completed with a physiotherapist. Subsequent training with a respiratory training device will be completed at 30% of the maximum value you achieved during the MEP examination. If you were not able to handle this set value during practical instruction by a physiotherapist for various objective or subjective reasons, you will complete the training with a lower value than the set 30% MEP. The physiotherapist told you the initial value that you will observe during the training of the exhalation muscles during the initial education.

You should observe the frequency and duration of expiratory muscle training during the rehabilitation program with a frequency of at least 5 times a week and a duration of each of the sessions at least 10 minutes, provided that you have completed the set number of sets and repetitions below.

Each of your respiratory training sessions will consist of 5 series of 10 repetitions. Take 1-minute break after each series.

Perform exhalation muscle training with an exercise machine sitting upright. When training the exhalation muscles, you do not need to wear a nasal clip, provided that you inhale through your nose or mouth, but only exhale through your mouth through Threshold® PEP. If you are not able to ensure this during training, we recommend the use of a nose clip. Exhale against the set resistance of the training device with a longer duration compared to the breath, their approximate ratio is 2.5-3: 1. The breath should not be maximal. Perform the respiratory training of the expiratory muscles in an upright sitting position and, if possible, with a visual inspection for better performance of your training (eg a mirror).

Exercise of oropharyngeal muscles

Exercises of the oropharyngeal muscles, which contribute to the partial or complete closure of the airways by their activity, involve exercises of the tongue, soft palate, face, neck

and jaws. You will perform this exercise according to your abilities in the home environment according to the entrance education by a physiotherapist and in compliance with the following parameters - frequency, duration, listed exercises and conditions of their implementation.

You should follow the frequency of the exercises, with the exercises listed below, at least with a frequency of 5 times a week and the duration of each of them at least 10 minutes, provided that you have practiced all the listed exercises, their number of repetitions and series.

The individual components of oropharyngeal muscle exercise have their own number of series and the number of repetitions, these must be observed. For individual exercises, there is a verbal description of their implementation, including any time information that must be observed. Perform all the above exercises in an upright sitting position and, if possible, exercise with a visual inspection of their performance (eg a mirror).

Tongue exercises (2 series, 12 repetitions)

- Stick the tongue out of the mouth towards the nose for 3 seconds.
- Stick the tongue out of the mouth towards the chin for 3 seconds.
- Press the back of the tongue against the base of the oral cavity while keeping the tip of the tongue in contact with the lower incisors for 3 seconds.
- Push the front half of the tongue onto the hard palate for 3 seconds.
- Suck the tongue up on the hard palate while pressing it against the hard palate for 3 seconds.

Soft palate exercises (2 series, 10 reps)

- Say the oral vowels "A, E, I, O, U" intermittently for 3 seconds.
- Say the oral vowels "A, E, I, O, U" continuously for 3 seconds.
- Inhale through your nose, exhale through your mouth with an extended exhale with your lips pressed together.

Face, Neck and Jaw Exercises (2 series, 10 reps)

- With your head slightly tilted back, swallow with your tongue slightly pressed between your teeth.
- Only perform facial suction movements with pouting lips.
- Press your lips together for 3 seconds.
- With your mouth wide open, pout your lips for 3 seconds.
- With your hand under your chin, try to open your mouth for 3 seconds, your hand preventing it from opening.

Teleconsultation - Telemonitoring, telecoaching and motivation

Regular teleconsultations, including telemonitoring, telecoaching, or motivation, will, according to prior agreement with the physiotherapist, take place by telephone or e-mail at least once every 5 days.

Telemonitoring for the purpose of analysis, evaluation and subsequent feedback of completed training and the course of the training program provides important information not only to the physiotherapist, but also to the patient with regard to the following training and further progress in the training program.

Telecoaching concerns the following trainings and the subsequent course of the program in the coming days before the next teleconsultation. The purpose of telecoaching is primarily to adjust your trainings or program according to the information obtained from the previous course of the program and trainings.

Motivation, as a possible important part of teleconsultations, can be an essential part of the whole program, thanks to which you will not give up your training efforts and persevere not only in regularly conducted set rehabilitation program, but even in long-term implementation of acquired habits in connection with the completed program.
